# Brugada syndrome: a fatal disease with complex genetic etiologies – still a long way to go

**DOI:** 10.1080/20961790.2017.1333203

**Published:** 2017-07-05

**Authors:** Yeda Wu, Mei Ai, Adham Sameer A. Bardeesi, Lunwu Xu, Jingjing Zheng, Da Zheng, Kun Yin, Qiuping Wu, Liyong Zhang, Lei Huang, Jianding Cheng

**Affiliations:** a Department of Forensic Pathology, Zhongshan School of Medicine, Sun Yat-Sen University, Guangzhou, China; b Forensic Science Center of WASTON Guangdong Province, Guangzhou, China; c The Branch Office of Yanping, Public Security Bureau of Nanping, Nanping, China

**Keywords:** Brugada syndrome, prevalence, genetics, gene variants, sudden death

## Abstract

Brugada syndrome (BrS) is an arrhythmogenic disorder which was first described in 1992. This disease is a channelopathy characterized by ST-segment elevations in the right precordial leads and is susceptible to sudden death. BrS is a fatal disease with gender and age preferences. It occurs mainly in young male subjects with a structurally normal heart and silently progresses to sudden death with no significant symptoms. The prevalence of BrS has been reported in the ranges of 5–20 per 10 000 people. The disease is more prevalent in Asia. Nowadays, numerous variations in 23 genes have been linked to BrS since the first gene *SCN5A* has been associated with BrS in 1998. Not only can clinical specialists apply these discoveries in risk assessment, diagnosis and personal medicine, but also forensic pathologists can make full use of these variations to conduct death cause identification. However, despite the progress in genetics, these associated genes can only account for approximately 35% of the BrS cases while the etiology of the remaining BrS cases is still unexplained. In this review, we discussed the prevalence, the genes associated with BrS and the application of molecular autopsy in forensic pathology. We also summarized the present obstacles, and provided a new insight into the genetic basis of BrS.

## Introduction

In November 1992, Pedro Brugada and Josep Brugada identified a hereditary disorder with a cardiac abnormality characterized by a distinct electrocardiogram (ECG) pattern, and a structurally normal heart [1]. By 1996, the Japanese cardiovascular researchers named the unique ECG findings as “Brugada Syndrome” (BrS) [2]. In 1998, a publication in *Nature* first linked *SCN5A* gene to BrS, opening a new era to explore the genetic defects in BrS [3]. In 2002 and 2005, two conference reports [4,5] focused on diagnostic criteria, risk stratification schemes, and approaches to therapy of BrS were published, providing a consensus understanding about the disease.

Generally speaking, BrS is a cardiac arrhythmia, characterized by ST segment elevation in leads V1–V3 with right bundle branch block in ECG [4]. BrS is associated with a high risk of sudden death, accounting for 20% of patients with structurally normal hearts [6]. The majority of BrS patients are asymptomatic, while others may suffer from syncope or even develop sudden death due to the fast polymorphic ventricular tachycardia. These symptoms may occur mainly after a large meal or during sleep or rest [7]. There is an obvious gender difference in BrS, approximately 80% of the patients are males. The age of these patients ranges from 1 to 84 years, but it mostly occurs around the age 40–45 [8].

To date, several genes are reported to be associated with BrS. However, the etiology of BrS remains unclear. This review discussed the epidemiology, reported genes associated with BrS, the role of molecular autopsy in forensic identification and the hypothesis of possible etiology of BrS.

## Epidemiology

It is difficult to determine the prevalence of BrS because the majority of these patients are asymptomatic, and in some patients, the first manifestation could be sudden death. The current estimation is based on ECG signs, which gives a rough estimation of the prevalence of the BrS [9–30]. Prevalence data of the BrS ECG pattern in different parts of the world is illustrated in Table 1. We concluded that the combined prevalence of BrS ECG is low with an incidence rate of 30 per 10 000 subjects. However, the prevalence of BrS may be lower than that due to its diagnostic strict criteria. Globally, this prevalence varies from 5 to 20 per 10 000 [8], and the syndrome appears to have a higher prevalence in Asia, which is illustrated in Table 2. Further population studies are needed to get more information about the prevalence of BrS.

**Table 1.** Reported BrS incidence in different area.

Continent	Area	Population	Total number of BrS^a^	Incidence(%)^b^	Ref.	Characteristics of population
Asia	Iran	3 895	100	2.57	[9]	54% women, mean age (38.2 ± 11.9) years
	Israel	592	5	0.84	[10]	346 men, 246 women; age 14–67 years
	Japan	8 612	12	0.14	[11]	5 987 men and 2 625 women, mean age 49.2 years
		8 006	23	0.29	[12]	Japanese-American men aged 45–68 years
		3 339	69	2.07	[13]	2 646 men and 693 women, age >18 years
		13 904	98	0.70	[14]	Mean age (58 ± 10) years
	Korea	10 867	98	0.90	[15]	10 867 men
	Pakistan	1 100	9	0.82	[16]	712 men and 388 women
	Philippines	3 907	94	2.41	[17]	–
	Taiwan	20 562	26	0.13	[18]	5 752 men and 14 810 women, mean age (49 ± 21) years
	Turkey	1 238	6	0.48	[19]	671 men, 567 women, mean age (38.9 ± 17.6) years
Europe	Austria	47 606	1	0.00	[20]	47 606 young Austrian men, median age 18 years
		4 491	26	0.58	[20]	4 491 patients with arrhythmia
	Denmark	18 974	13	0.07	[21]	-
	Finland	2 479	15	0.61	[22]	2 479 young men, age 18–30 years
		542	3	0.55	[22]	542 healthy subjects, 274 men and 268 men, age 40–60 years
	France	35 309	11	0.03	[23]	47% men, mean age 37.2 years
		1 000	61	6.10	[24]	63% men, mean age 39
	Germany	4 149	0	0.00	[25]	Aged 25–74 years
	Greece	11 488	25	0.22	[26]	58% men, aged 15–98 years
	Italy	12 012	31	0.26	[27]	10 901 men, mean age (29.9 ± 9) years
North America	USA	12 000	52	0.43	[28]	–
		27 328	18	0.07	[29]	–
		162 590	456	0.28	[30]	–

a: Including BrS patients and BrS ECG pattern.

b: Incidence = (Total number of BrS/Population) × 100.

**Table 2.** BrS incidence in different continent.

Continent	Population	Total number of BrS^a^	Incidence (%)^b^
Asia	76 022	540	0.71
Europe	138 050	186	0.38
North America	201 928	526	0.26
Total	415 990	1 252	0.30

a: Including BrS patients and BrS ECG pattern.

b: Incidence = (Total number of BrS/Population) × 100.

## Genetic factors of BrS

Ion channel, located on the membrane of the cardiomyocytes, is protein which allows ions to move in and out to make an ion balance. During the process of channel opening and closing, the cardiac action potential is produced [31]. To date, studies have identified many genetic factors in cardiac sodium, potassium and calcium channels associated with BrS. The currently reported BrS-related genes are illustrated in Table 3.

**Table 3.** Reported gene associated with BrS.

Component of ionic current	Gene name	Locus	Gene product	Mutation functional effect
Sodium	*FGF12*	3q28–q29	Fibroblast growth factor 12	↓I_Na_
	*GPD1-L*	3p22.3	GPD1-L	↓I_Na_
	*HEY2*	6q22.31	Hey2	↓I_Na_
	*PKP2*	12p11.21	Plakophillin-2	↓I_Na_
	*RANGRF*	17p13.1	MOG1	↓I_Na_
	*SCN1B*	19q13.11	Na_V_β1	↓I_Na_
	*SCN2B*	11q23.3	Na_V_β2	↓I_Na_
	*SCN3B*	11q24.1	Na_V_β3	↓I_Na_
	*SCN5A*	3p22.2	Na_V_1.5	↓I_Na_
	*SCN10A*	3p22.2	Na_V_1.8	↓I_Na_
	*SLMAP*	3p14.3	Sarcolemma-associated protein	↓I_Na_
Calcium	*CACNA1C*	12p13.33	Ca_V_1.2	↓I_Ca_
	*CACNB2b*	10p12.33–p12.31	Ca_V_β2b	↓I_Ca_
	*CACNA2D1*	7q21.11	Ca_V_α2δ	↓I_Ca_
	*TRPM4*	19q13.33	TRMP4	↓/↑NSC_Ca_
Potassium	*ABCC9*	12p12.1	SUR2A	↑I_K-ATP_
	*KCND3*	1p13.2	K_V_4.3	↑I_To_
	*KCNE3*	11q13.4	MiRP2	↑I_To_/I_Ks_
	*KCNE5*	Xq23	MiRP4	↑I_To_/I_Ks_
	*KCNH2*	7q36.1	hERG2	↑I_Kr_
	*KCNJ8*	12p12.1	Kir6.1	↑I_K-ATP_
	*SEMA3A*	7q21.11	Semaphorin	↑I_To_
Mixed	*HCN4*	15q24.1	HCN4	--/I_f_

### Gene encoding sodium channels in BrS

Cardiac sodium channel, consisting of a pore-forming α-subunit and several regulatory β-subunits, plays a very important role in the generation of the rapid upstroke of an action potential and the transmission of cardiac impulsion.

#### FGF12

This gene, located at 3q28–q29, encodes one of the fibroblast growth factor (FGF) homologous factors (FHFs). The FHFs genes are supposed to be arrhythmogenic loci due to the nature that FHFs have the ability to affect both sodium and calcium currents [32]. On the basis of the fact mentioned above, *FGF12*, the major FHF expressed in the human heart ventricles, was detected in 102 unrelated patients with BrS by Hennessey et al. [32]. The study showed a missense mutation Q7R, of which the function can reduce sodium current through affecting Na^+^ channel trafficking, suggesting that *FGF12* may underlie the pathogenesis of BrS.

#### GPD1-L

In 2002, Weiss et al. [33] described a novel BrS-susceptibility gene locus in a large multi-generational family of Italian descent. This gene is located on 3p22.3, of which the position is nearby the *SCN5A* gene. After 5 years, London et al. [34] identified the gene as *GPD1-L* gene, which encodes the glycerol-3-phosphate dehydrogenase 1-like protein. Using direct sequencing, London et al. found a missense mutation (A280V) in the gene. Compared with WT *GPD1-L* and WT *SCN5A*, the co-expression of A280V *GPD1-L* with WT *SCN5A* led to a reduced inward Na^+^ current approximately by 50%, and a decreased *SCN5A* surface membrane expression, indicating that *GPD1-L* gene causes BrS through affecting the expression of *SCN5A*. As for the mechanism of how a mutation in *GPD1-L* gene influences Na_V_1.5, based on the homology between *GPD1-L* and *GPD*, Liu et al. [35] put forward a hypothesis that *GPD1-L* may serve a similar function as *GPD*, which involves in NAD-dependent energy metabolism. They also demonstrated that A280V *GPD1-L* can induce elevation of [NADH]i, which can downregulate I_Na_ acutely through a PKC activation and increased superoxide. The study suggests metabolism plays a role in I_Na_. Another study [36] showed that the *GPD1-L* mutation itself causes a loss function of enzymatic activity, and decreased *GPD1-L* activity would have then increased the substrate G3P, and fed the PKC-mediated phosphorylation of *SCN5A* at S1503 where such phosphorylation was known to decrease I_Na_. A study showed that *GPD1-L* gene accounted for 11%–12% BrS probands [37], but other studies showed no identification on any missense *GPD1-L* gene mutation, suggesting that *GPD1-L* may not be a major cause of BrS [38,39]. Further studies are needed to obtain the accurate prevalence of *GPD1-L* variants in BrS.

#### HEY2

Located at 6q22.31, the gene encodes Hey2 – a basic helix–loop–helix transcriptional repressor which is expressed in the cardiovascular system [40]. Bezzina et al. [41] identified a mutation rs9388541, located downstream of the gene, in 312 individuals with BrS through a genome-wide association study. The gene, supported by the evidence that *HEY2* regulates cardiac electrical activity, is associated with the pathogenesis of BrS.

#### PKP2

*PKP2* gene is located at 12p11.21, and encodes the desmosomal protein plakophilin-2, which is the major genetic cause of arrhythmogenic right ventricular cardiomyopathy (ARVC) [42]. It is reported that plakophilin-2 is associated with sodium channel, of which absence can decrease I_Na_ [43]. Cerrone et al. [44] analyzed *PKP2* variants in 200 BrS patients, and found five amino acid substitutions (Q62K, S183N, T526A, R635Q, and M365V) which can result in loss of sodium channel function, indicating that *PKP2* may be a molecule substrate of BrS. Cerrone et al. [44] proposed that reduced *PKP2* expression could modify the interaction of Na_V_1.5 with other partners that impact on gating properties.

#### RANGRF

The *RANGRF* gene, also known as *MOG1* gene, is located at 17p13.1, which encodes MOG1 protein (a co-factor of Na_V_1.5). Expression of *MOG1* in Na_V_1.5-expressing cells increased I_Na_ without changes in biophysical properties of the channel [45]. Kattygnarath et al. [46] identified a missense mutation E83D in *RANGRF* gene. Expression of this mutation failed to increase I_Na_, demonstrating the mutant exerts a dominant negative effect on WT-*MOG1*. The studies mentioned above suggest that the mutation in *RANGRF* gene may cause BrS by reducing Na_V_1.5 channel trafficking to the cell surface, and *RANGRF* is a new susceptibility gene for BrS. In two subsequent studies, both Olesen et al. [47] and Campuzano et al. [48] uncovered another *RANGRF* variant E61X in 220 Danish patients, including 197 AF patients, 23 BrS patients and a Spanish family. This variant caused a complete loss function of *MOG1*, thus eliminating the I_Na_, and conferring a loss of function of I_Na_. Based on the mechanism of *MOG1* interaction with Na_V_1.5, Chakrabarti et al. [49] supposed that use of *MOG1* to enhance Na_V_1.5 trafficking to plasma membrane may be a potential personalized therapeutic approach for some patients with BrS in the future.

#### SCN5A

The first gene associated with BrS is *SCN5A*, located at chromosome 3p22.2, which encodes the α-subunit of the voltage-dependent cardiac sodium channel, Na_V_1.5. In 1998, Chen et al. [3] described the mutations in *SCN5A* which is relevant to BrS, since then more than 300 BrS-related mutations in *SCN5A* have been found. It is accepted that *SCN5A* represents the major gene in BrS pathogenesis, and mutations in the *SCN5A* gene account for approximately 18%–30% of BrS cases [50]. Many different *SCN5A* mutations have been studied in a various expression systems, and the results lead to a reduction in the cardiac sodium current. Actually, several studies have been performed to demonstrate that mutations in *SCN5A* such as R1432G [51], G1743R [52] and T353I [53] give rise to defect trafficking of the channel. Hsueh et al. [54] and Calloe et al. [55] characterized mutations in *SCN5A*, and concluded that the mutations can reduce the current via a shift in the sodium channel current activation, inactivation or a closed state. There are also several manuscripts that focused on the intermediate state of inactivation, from which it recovers slower than normal [56,57]. In other words, the defects of sodium channel caused by mutations in *SCN5A* are diverse, and mutations in *SCN5A* gene play a key role in the pathogenesis of BrS.

#### SCN10A

This gene, located at 3p22.2, encodes a neuronal sodium channel Na_V_1.8 which is principally involved in nociception [58]. Na_V_1.8 was reported to modulate SCN5A expression and cardiac electrophysiology in heart [59], and genome-wide association study (GWAS) showed that single nucleotide polymorphisms in *SCN10A* were associated with cardiac conduction defects, including rs6795970, rs6798015, rs6800541 and rs7430477 [60], suggesting that *SCN10A* plays an important role in cardiac arrhythmia. Based on its role in arrhythmogenesis, Hu et al. [61] screened *SCN10A* variants in 150 BrS patients, and identified 17 mutations in 25 probands. Functional study showed that co-expression of *SCN5A*/WT and *SCN10A*/WT led to a doubling gain of I_Na_ in contrast to co-expression of *SCN5A*/WT and *SCN10A*/(R14L, and R1268Q). The obviously decreased I_Na_, together with the identification of *SCN10A* in 16.7% of BrS probands, indicates *SCN10A* as a major susceptibility gene for BrS. Recently, Fukuyama et al. [62] identified 5 *SCN10A* variants (W189R, R844H, N1328K, R1380Q and R1863Q) in 6 out of 240 BrS probands, although the functional significance of these variants remains unclear, stressing an important role of *SCN10A* in BrS.

#### SCN1B

The *SCN1B* gene encodes two isoforms of β1-subunit, β1 and β1b, the former arises from splicing of exons 1–5 of the gene, and the latter arises from splicing of exons 1–3 with retention of a segment of intron 3, both of which are expressed through the heart [63]. In 2001, Isom reported the function of β subunit, including an increase in Na_V_1.5 expression on the cell surface, modulation of channel gating, and voltage dependence, and playing a role in cell adhesion, and recruitment of cytosolic proteins such as Ankyrin-G [64]. In 2008, Watanabe et al. [65] investigated *SCN1B* variants in 282 probands with BrS, and identified one mutation (W179X) in a patient with BrS, which can cause a reduced Na_V_1.5 sodium current as a result of loss or altered β-subunit modulation of Na_V_1.5 current. Hu et al. [66] detected an R214Q variant in *SCN1Bb*. When co-expressed with *SCN5A*/WT, and *KCND3*/WT separately, the variant induced a decrease in the peak sodium current, and a greater I_To_, providing another mechanism that a combined loss of function of I_Na_ and a gain function of I_To_ are responsible for BrS pathogenesis. Lin et al. [67] proposed that life-threatening arrhythmias in patients with mutations in *SCN1B* gene can be partly consequent to be disrupted intracellular Ca^2+^ homeostasis. There were several studies reporting the *SCN1B* gene mutation variants among BrS patients, including W15Y, R124G, A197D and H162P, suggesting that the occurrence of *SCN1B* gene variants in BrS is not rare [68,69].

#### SCN2B

This gene encodes the β_2_-subunit of the cardiac sodium channel, and was linked to BrS by Riuro et al. [70] in 2013. They found a novel missense mutation D211G in a woman diagnosed with BrS. Compared with cells co-expressing *SCN5A*/WT+*SCN2B*/WT, a 39.4% reduction of I_Na_ was observed within the cells co-expressing *SCN5A*/WT+*SCN2B*/D211G without any changes on unitary channel conductance, suggesting that D211G reduced I_Na_ by decreasing Na_V_1.5 cell surface expression. As for the incidence of BrS in this gene, Koopmann et al. [71] reported no mutation in *SCN2B*, and concluded that *SCN2B* is rare in BrS.

#### SCN3B

Morgan et al. [72] identified this gene in 2000. *SCN3B* is located at 11q24.1, which encodes the β3-subunit, Na_V_β3. The β3-subunit plays a role in making subtle changes to the sodium channel gating. The gene was first linked to BrS by Hu et al. [73], who identified a novel mutation (L10P) of *SCN3B* in a male with BrS. Compared to *SCN5A*/WT+*SCN1B*/WT+*SCN3B*/WT, the missense mutation co-expressed with *SCN5A*/WT+*SCN1B*/WT induced a decrease in peak sodium current density, accelerated inactivation, and slowed reactivation, causing a loss function of I_Na_. Another mutation V110I was identified in 3 of 178 (1.7%) Japanese BrS patients [74], and the functional study showed this mutation led to a reduced sodium current by impairing the cytoplasmic trafficking of Na_V_1.5. The two studies above suggest the *SCN3B* gene plays a role in the pathogenesis of BrS.

#### SLMAP

*SLMAP*, located at 3p14.3, encodes the sarcolemmal membrane-associated protein. This protein is a component of T-tubules and sarcoplasmic reticulum, of which the functional association can regulate the excitation of cardiomyocytes [64]. Ishikawa et al. [75] identified two missense mutations (V269I and Q710A) in 190 unrelated BrS patients. The functional study showed that the mutations reduced expression of Na_V_1.5 on the cell surface by impairing the trafficking process of Na_V_1.5, thus resulting in a decreased sodium current.

### Gene encoding calcium channels in BrS

Human L-type voltage-gated calcium channel (LTCC), also known as Ca_V_1.2, consists of a complex of α_1_, α_2_δ, β and γ subunits in a 1:1:1:1 ratio, which mediates the influx of calcium ions into the cell upon membrane polarization. Each protein mentioned above has multiple isoforms as a result of alternative splicing.

#### CACNA1C

*CACNA1C* is located at 12p13.33 and encodes the α1 subunit of Ca_V_1.2 [76]. In 2007, Antzelevitch et al. [77] identified two missense mutations in two out of 82 BrS probands, including G490R and A39V. Functional studies revealed that both mutations led to a loss-of-function in calcium channel activity, and the displayed loss-of-function effect induced by A39V was found to be caused by a trafficking defect. Since then, the gene has been associated with BrS. In 2014, Beziau et al. [78] found a missense mutation N300D in four out of a family with five BrS patients, and expression of N300D in COS-7 cells led to a loss-of-function in Ca_V_1.2. Besides, a global expression defect of the mutant Ca_V_1.2 and an increased mobility of the mutant Ca_V_1.2 were observed, suggesting an acceleration of Ca_V_1.2 turnover by destabilization which may lead to a quicker degradation of the protein. In another study [79], a novel mechanism emphasized that the role of splicing mutation in *CACNA1C* and the nonsense-mediated mRNA decay (NMD) can lead to a decrease in mutant mRNA, suggesting it to be associated with the pathogenesis of BrS.

#### CACNB2b

This gene, located at 10p12.33-p12.31, encodes the β-subunit of Ca_V_1.2, also known as Ca_V_β_2_, which is involved in regulation and intracellular trafficking of L-type calcium channel current (I_Ca-L_) [80]. Anzelevitch et al. [77] found a heterozygous C1442T transition in exon 13, causing a substitution of leucine for serine at position 481 (S481L) of *CACNB2b*, in one proband with BrS. Genetic testing was performed on the family members of this proband, and the S481L mutation was present in all six phenotype-positive, but absent in all four phenotype-negative family members. Expression of this mutation revealed a markedly reduced I_Ca-L_. As the mutation is located in close proximity to the DI–DII linker of Ca_V_1.2, interference with the stimulatory role of Ca_V_β_2_ on I_Ca-L_ is a likely pathogenic mechanism for this mutation [81]. In 2009, Cordeiro et al. [82] uncovered a missense mutation T11I in *CACNB2b* resulting in accelerated inactivation of LTCC. Subsequently, the accelerated inactivation of I_Ca-L_ translated into reduced depolarizing current predisposing to the development of BrS. Several other mutations have been found in *CACNB2b* that is associated with BrS [83,84], of which the functional studies are needed to be performed to demonstrate the pathogenic mechanism of these mutations.

#### CACNA2D1

This gene, located at 7q21.11, encodes the α_2_δ-subunit of the Ca_V_1.2, and has been found to share a similar functional property with Ca_V_β_2_ [85]. Burashnikov et al. [83] reported *CACNA2D1* may be a BrS susceptibility gene on the basis of the fact that three different missense mutations (S709N, D550Y and Q917H) were identified in *CACNA2D1* in three BrS patients out of 205 patients with BrS, short QT, idiopathic ventricular fibrillation and early repolarization syndrome (ERS). However, further functional studies are needed to certify whether this gene is associated with BrS, and to understand more about the role of these mutations in BrS.

#### TRPM4

The transient receptor potential melastatin protein 4 gene, also known as *TRPM4*, encodes a calcium-activated nonselective cation channel (NSC_Ca_), which has been implied with progressive cardiac conduction blocks  [86]. Since BrS is frequently associated with cardiac conduction abnormalities, Liu et al. [87] screened *TRPM4* in 248 BrS cases without *SCN5A* mutation, and found 11 mutations, of which five were absent from control alleles, and four were statistically more prevalent than in control alleles. They further studied four selected mutations, and revealed these mutations can result in both a gain-of-function (P779R and K914X) and a loss-of-function (T873I and L1075P) of NSC_Ca_. However, further studies are needed to demonstrate its role in the pathophysiology of BrS.

### Gene encoding potassium channels in BrS

The potassium channel represents the most complex class of ion channels from both functional and structural standpoints [88]. The ion-conducting pore of a K^+^ channel is formed by four α-subunits that co-assemble as homo- or heterotetramers with different biophysical properties. Their gating characteristics are further modulated by ancillary subunits. The potassium channels can be divided into different types, for instance, voltage-gated potassium (K_V_) channels (I_To_, I_Ks_) and inward-rectifier type potassium channel (I_K1_, I_K-ACh_, I_K-ATP_, I_Kr_) [89].

#### ABCC9

ATP-sensitive potassium cardiac channels (I_K-ATP_) consist of inward rectifying channel subunit Kir6.1 or Kir6.2, and the sulfonylurea receptor subunits SUR2A. *ABCC9*, located at 12p12.1, encodes the ATP-binding cassette transporter of I_K-ATP_ (SUR2A). Hu et al. [90] identified several mutations in 150 BrS or ERS probands. However, it remains unclear whether these mutations can be considered a causality for BrS. Further studies are needed to confirm the role of *ABCC9* in BrS.

#### KCND3

I_To,f_ (fast transient outward potassium current), a voltage-gated potassium channel expressed in heart, is a complex that consists of four α-subunits (encoded by *KCND3*, K_V_4.3) and two β-subunits (K^+^ channel interacting protein 2, KChIP2). In 2011, Giudicessi et al. [91] identified two novel mutations (L450F and G600R) in two out of 86 unrelated BrS patients. Compared with *KCND3*/WT+KChIP2/WT, co-expression of *KCND3*/(L450F or G600R)+KChIP2/WT revealed a significant increase in I_To,f_. Subsequently, the mutations (L450F and G600R) caused a gain-of-function of I_To,f_ through increasing membrane protein expression, and slowing channel inactivation is demonstrated [92].

#### KCNE3

I_Ks_ (slowly activated delayed rectifying potassium current) is composed of four α-subunits (encoded by *KCNQ1*, K_V_7.1) and two β-subunits (encoded by *KCNE1*, Mink). *KCNE3*, located at 11q13.4, is a member of five *KCNE* genes, of which the encoding product MiRP2 is involved in modulating the function of cardiac potassium currents, for example, I_To_ and I_Ks_ [89,93]. Delpon et al. [93] first identified the association between *KCNE3* and BrS. They found an R99H missense mutation in one male out of 105 probands with BrS. The results of gene testing on the family of this individual showed that 4/4 phenotype-positive and 0/3 phenotype-negative family members had the mutation. They also demonstrated that K_V_4.3 and *KCNE3* can be co-immunoprecipitated. Compared with *KCND3*/WT+*KCNE3*/WT, co-expression of *KCNE3*/R99H and *KCND3*/WT led to a significant increase in peak current and an accelerated inactivation of I_To_. Not only did the study show that *KCNE3* had a modulation of I_To_, but also suggested that the mutation in *KCNE3* underlies the development of BrS. Nakajima et al. [94] also identified a mutation T4A in one out of 40 BrS ECG-pattern patients with an increased I_To_.

#### KCNE5

This gene, one of five *KCNE* members (*KCNE1-5*), encodes MiRP4 which is one of the regulatory β-subunits of I_Ks_. Besides, it has been reported that β-subunits encoded by *KCNE* family affect I_To_. Ohno et al. [95] identified two novel variants (Y81H in three probands and [D92E; E93X] in one proband) from 205 Japanese BrS or IVF patients without *SCN5A* mutation. Both the four probands were from four unrelated families. They identified 11 mutant carriers in the four families: three men and eight women, and all the males were symptomatic whereas six of eight women remained asymptomatic; this can be explained by the fact that *KCNE5* is located on chromosome X, and the upregulation of I_To_ currents may occur preferentially in male (XY) than in female mutation carriers (XX), which underlines the gender difference of BrS. In contrast, Y81H was identified in three women and [D92E; E93X] was absent from 300 unrelated healthy Japanese controls. Compared with *KCND3*/WT+*KCNE5*/WT, co-expression of *KCND3*/WT+*KCNE5*/ (Y81H or [D92E; E93X]) led to a gain-of-function of I_To_, indicating the clear *KCNE5* association with BrS.

#### KCNH2

*KCNH2*, also known as the human ether-a-go-go (hERG) gene, encodes an α-subunit of the I_Kr_, which plays an important role in regulating the repolarization of the cardiac action potential. In 2005, Verkert et al. [96] identified two mutations (G873S and N985S) in two out of 78 unrelated *SCN5A* mutation-negative BrS patients. Both mutations led to an increased I_Kr_ density and caused a negative shift of voltage-dependent inactivation, resulting in increased rectification. This increased density in I_Kr_ enhanced the susceptibility of loss of action potential dome in the right ventricular subepicardial myocytes, which contributes to the BrS phenotype. Given the glycine at position 873 of the hERG channel is not conserved between human, mouse and rat hERG proteins, along with the fact that G873S was also found at a heterozygous frequency of 0.4% in 500 unrelated Han Chinese controls, the *KCNH2* gene is thought to play a modifying role in BrS. Subsequently, several *KCNH2* mutations, including T152I, R164C, W927G and R1135H, which exert gain-of-function of I_Kr_, have been identified in probands with BrS [97–99].

#### KCNJ8

This gene, located at 12p12.1, encodes Kir6.1, an inward rectifying channel subunit involved in both ventricular and atrial repolarization [100]. Medeiros-Domingo et al. [101] found a *KCNJ8* missense mutation S422L in one out of 87 BrS patients, and functional investigation revealed that this mutation led to a gain-of-function of I_K-ATP_. The same missense mutation was also found in three other BrS patients by Barajas-Martinez et al. [102]. The whole cell patch clamp studies showed a two-fold gain of function of glibenclamide-sensitive I_K-ATP_ when *KCNJ8*/S422L was co-expressed with SUR2A/WT. Besides, it has been also demonstrated that S422L-induced gain-of-function in I_K-ATP_ is due to reduced sensitivity to intracellular ATP.

#### SEMA3A

This gene, located at 7q21.11, encodes semaphorin 3A, a chemorepellent which involved in both neural and cardiac innervation patterning. It has been demonstrated that semaphorin 3A is a naturally occurring protein inhibitor of K_V_4.3 [92]. When co-expressed with K_V_4.3, *SEMA3A* altered K_V_4.3 by significantly reducing peak current density. Besides, co-immunoprecipitations of *SEMA3A* and K_V_4.3 revealed a potential direct binding interaction between these proteins. Nicole et al. [103] identified two rare missense *SEMA3A* mutations in 198 unrelated BrS patients with non-*SCN5A* mutation; on the cellular basis, these mutations disrupted the ability of semaphorin 3A to inhibit K_V_4.3, and resulted in a significant gain of I_To_ compared with *SEMA3A*/WT, indicating a novel susceptibility of the *SEMA3A* gene for the pathogenesis of BrS.

### Gene encoding mixed ion channel in BrS

#### HCN4

*HCN4*, located at 15q24.1, of which the encoding product is hyperpolarization-activated cyclic nucleotide-gated channel 4 protein. This protein forms the tetramer complex of the human cardiac pacemaker channel of which the ion current is the funny current. The funny current is made up of two ion component: sodium and potassium. In 2012, Crotti et al. [84] identified an *HCN4* mutation S841L in one out of 129 unrelated BrS possible patients, which was absent in 1 400 Caucasian reference allele. It has been reported that *HCN4* expresses at a very low level in the ventricular myocardium, indicating an indirect role in causing BrS. However, further studies are needed to assess the functional effect of the mutation and to investigate whether this gene plays a role in the BrS pathogenesis.

## The role of post-mortem genetic testing in the forensic identification

In forensic pathology, the whole process of an autopsy at present includes gross examination and dissection, microscopic histopathological examination and different laboratory tests (toxicologic analysis and microbiological studies). These procedures can meet most of the requirements of death cause identification. However, there remain some cases, the death of which occurs within one hour without a clear cause of death after death scene investigation, systematic autopsy, histopathological examination and laboratory test. These cases are defined as sudden unexplained death (SUD) cases. SUD remains a difficulty in forensic identification and it will be greatly helpful for the development of forensic pathology if the death causes of SUD can be figured out.

As the genetic technologies are making progress, the death causes of parts of these SUD cases can be explained by using post-mortem genetic testing (also named “molecular autopsy”). The appraiser find that the death causes of parts of these SUD are cardiac arrhythmias and cardiomyopathies caused by pathogenic genetic variants, such as BrS. So it is necessary to identify the genetic alterations in cardiac arrhythmias and cardiomyopathies responsible for SUD cases. Bagnall et al. [104] performed postmortem genetic testing on a cohort of 50 consecutive SUD cases using exome sequencing, and 9 variations in 27 common genes of cardiac arrhythmias and cardiomyopathies were identified in nine out of a subgroup of 28 SUD cases. Among the nine variations, one variation each was identified in *CACNA2D1* and *CACNA1C* (both are BrS-associated genes). The exome sequencing-based molecule autopsy is a useful strategy for the investigation of SUD cases. Sudden unexplained nocturnal death syndrome (SUNDS) is a kind of SUD with unclear etiology, which is thought to be a genetically, phenotypically and functionally the same allelic to BrS [105]. Twenty-two out of 40 SUNDS victims were reported to carry 40 variants in 80 genes associated with arrhythmia/cardiomyopathy using molecular autopsy in the study of Zhang et al. [106]. Among the 40 variants, five variants in four BrS-associated genes (*SCN5A*, *SCN1B*, *CACNA1C* and *CACNB2b*) were identified. The study suggests that post-mortem molecular autopsy is helpful in explaining the death cause of these SUD cases. The two studies mentioned above also provide an insight to search the potential pathogenic gene associated with arrhythmia and cardiomyopathy for SUD cases. Thus, understanding the pathogenesis (especially, genetic component) of each cardiac arrhythmia and cardiomyopathy is useful for forensic pathologist to identify the death cause of part of SUD.

However, nowadays, most of the data regarding molecular autopsy are based on laboratory research. The cost of molecular autopsy is expensive and this tool requires more strict preservation condition for the victim's blood and tissue. Due to these limitations, this tool cannot be widely used at present. But molecular autopsy should be mature enough in the near future to be widely applied as a forensic routine tool in death cause identification.

## Possible explanation for the etiology of BrS

Despite the fact that a number of variants have been uncovered, it has been estimated that nearly 30% carry a pathogenic variant in *SCN5A* gene, and other identified genes presently together are responsible for 5% of all BrS cases. Sixty-five percent of BrS cases do not have a clear explanation of the etiology. There are several possible explanations as follows: (1) there are other potential genetic mechanisms which are not clear yet, such as copy number variations described in *SCN5A* [107]; (2) pathogenic variants associated with BrS are located in unknown genes; (3) other factors, which may affect the process of transcription and translation, such as DNA methylation, post-translational modifications and RNA mechanism [79]; (4) another issue should be taken into consideration is the complex inheritance of BrS. The disease has been regarded as a monogenic disease with autosomal dominant mode of inheritance, and many studies were mainly focused on seeking for new genes or genetic variants in individuals or families affected by BrS. However, the inheritance of BrS does not meet the criteria of Mendelian inheritance after analyzing genetic variants in large family pedigrees with BrS, and this phenomenon was described as low-penetrance or phenocopies. A recent study [108] showed that common polymorphic alleles strongly associated with disease risk, and stressed the importance of defining a proper genetic model for BrS.

## Perspective

BrS is a channelopathy characterized by a structurally normal heart leading to arrhythmogenesis, syncope and sudden death. The disease has an age and gender preference, and, as aforementioned, most death events occur in males in their forties. The syndrome is more prevalent in Asia, while most BrS deaths occur at rest or during sleep. Progress in genetics has helped both to unravel the origin of BrS and to understand the mechanistic pathways. Compared to nearly 20 years ago, nowadays mutations in 23 genes have been associated with BrS or BrS ECG phenotype. In most cases, the gene or the rare genetic variants (named as candidate genes) were speculated to play a pathogenic role when compared with in-house or public disease variants database, and then the putative-causing genes or variants were identified on the basis of the pathophysiological experiment from candidate genes [108]. However, the study design, the number of studied population and the functional data are different, and the strength of disease association needs further assessments [108]. Regarding this, the previously published “pathogenic” variants have been shown to be at a high minor allele frequency in general population [109], and thus its role in BrS needs to be re-assessed. At present, direct sequencing and functional studies still play an important role in the identification of pathogenic genes. Despite the complicated etiology of BrS, new genomic technologies, for example, GWAS and whole-genome next-generation sequencing, can be used to identify new candidate genes responsible for BrS. Besides, induced pluripotent stem cells (iPS) can be differentiated into cardiomyocytes, which provides a new insight for the electrophysiological and molecular study of BrS. In conclusion, the progress in genetics has provided a better understanding and facilitated the study of many diseases including BrS, and also contributed to the development of both forensic pathology and clinical cardiology. Continuing efforts in researching the etiology of disease is needed to help us understand the pathogenesis of BrS by new technologies.
